# Characterization and phase I study of CLR457, an orally bioavailable pan-class I PI3-kinase inhibitor

**DOI:** 10.1007/s10637-018-0627-4

**Published:** 2018-08-03

**Authors:** James J. Harding, Todd M. Bauer, Daniel S. W. Tan, Philippe L. Bedard, Jordi Rodon, Toshihiko Doi, Christian Schnell, Varsha Iyer, Fabienne Baffert, Rajkumar Radhakrishnan, Claire Fabre, Dejan Juric

**Affiliations:** 1 Memorial Sloan Kettering Cancer Center, New York, NY USA; 2 Sarah Cannon Research Institute / Tennessee Oncology, PLLC, Nashville, TN USA; 3 National Cancer Centre, Singapore, Singapore; 4 Princess Margaret Cancer Centre, Toronto, ON Canada; 5 Hospital Vall D’Hebron, Catalunia, Barcelona, Spain; 6 National Cancer Center East, Kashiwa, Chiba, Japan; 7 Novartis Pharma AG, Basel, Switzerland; 8 Agios Pharmaceuticals, Inc., Cambridge, MA USA; 9 Novartis Healthcare Pvt Ltd, Hyderabad, Telangana India; 10 Massachusetts General Hospital, 55 Fruit St, Boston, MA 02114 USA

**Keywords:** CLR457, Pan-PI3K inhibitor, Therapeutic index, Phase I, Preclinical

## Abstract

*Background* CLR457 is an orally bioavailable pan-phosphatidylinositol-4,5-bisphosphate 3-kinase (PI3K) inhibitor. *Methods* CLR457 anti-tumor activity and pharmacokinetics (PK) were characterized by in vitro biochemical assays and in vivo tumor xenografts. A first-in-human study was conducted to determine the maximum tolerated dose (MTD), safety, PK, and efficacy of CLR457. Successive cohorts of patients with advanced solid tumors with PI3K pathway activation received increasing CLR457 doses according to a Bayesian escalation model based on the rate of dose limiting toxicity (DLT) in the first 28-day cycle. *Results* CLR457 inhibited p110α, p110β, p110δ and p110γ isoforms with an IC_50_ of 89 ± 29 nM, 56 ± 35 nM, 39 ± 10 nM and 230 ± 31 nM, respectively. CLR457 exhibited dose-dependent antitumor activity and interfered with glucose homeostasis in PI3K-mutant tumor xenografts. 31 patients received doses ranging from 5 to 100 mg. DLTs included grade 3 hyperglycemia and rash (3). In the 100 mg cohort (*n* = 11), 3 (27.3%) patients had DLTs and all patients (100%) experienced ≥ grade 3 toxicity with rash (45.5%) as the most common event. The MTD was not determined. For the entire study population, stomatitis (45.2%), diarrhea (38.7%), rash (35.5%) were the most common any grade toxicities—51.6% patients experienced ≥ Grade 3 toxicity. CLR457 was rapidly absorbed with limited accumulation and linear PK. PK modeling indicated that pharmacologically active concentrations were achieved at the highest dose tested (100 mg), though no objective responses were observed. *Conclusion* CLR457 clinical development was terminated due to poor tolerability and limited antitumor activity. These results emphasize the difficulty of achieving a wide therapeutic index when targeting all class I PI3K-isoforms.

**Electronic supplementary material:**

The online version of this article (10.1007/s10637-018-0627-4) contains supplementary material, which is available to authorized users.

## Introduction

The PI3K/AKT/mammalian target of rapamycin (mTOR) pathway is a key intracellular signaling pathway, regulating critical cellular processes such as cell growth, proliferation, and survival. [1] PI3 kinases, a key component of this pathway, are a family of lipid kinases divided into three classes that differ in structure, preferred substrate, tissue distribution, mechanism of activation, and ultimately in function. [1] The class I PI3Ks functions as heterodimers consisting of one of four catalytic p110 subunits (p110α, β, δ or γ) and a regulatory subunit (p85α). [1] Genomic alterations in *PI3KCA, PTEN,* or other nodes in PI3K/AKT/mTOR pathway, contribute to oncogenesis in multiple cancers, [2–4] and pharmacologic interference with this pathway is deleterious to PI3K-addicted tumors in vitro and in vivo. Thus, the PI3K/AKT/mTOR pathway is an attractive target for anticancer therapy. [5, 6]

A myriad of PI3K/AKT/mTOR pathway inhibitors with unique molecular properties are in clinical development and have met with varying degrees of clinical success. [7] Rapalogues, the first effective inhibitor of this pathway, produce modest tumor shrinkage and improvement in outcomes in a number of solid tumors. [8–10] The preclinical observation that these agents induced key signaling proteins upstream of mTOR drove the development of both pan-PI3 kinase and dual PI3K/mTOR inhibitors. Although pan-PI3 kinase and dual PI3K/mTOR inhibitors should have widespread utility given the high proportion of alterations in *PI3KCA* and *PTEN* in human cancer, the issue of therapeutic index is of paramount importance for their drug development. The clinical experience with buparlisib, a selective pan-PI3K inhibitor (p110α: 52 nM; p110β: 166 nM; p110δ: 116 nM), validates the potential of pan-class 1 inhibition in advanced solid tumors, though highlights the importance of tolerability. [11–15] The toxicity profile of buparlisib is, in part, due to central nervous system (CNS) penetration and off-target effects on microtubules, and might be improved with the development of a novel pan-class 1 PI3K inhibitor without such effects. CLR457 is a potent, balanced pan-class 1 PI3K inhibitor designed to abrogate CNS penetration and microtubule destabilization (unpublished data). Here, we present the initial preclinical characterization of CLR457 as well as results from the first-in-human phase I study in patients with advanced solid tumors with PI3K pathway activation.

## Materials and methods

### Preclinical experiments

CLR457 was synthesized in the Global Discovery Chemistry group (Novartis). In vitro PI3K and protein kinase biochemical assays were performed as described previously to determine isoform-specific potency. [13] To evaluate antitumor activity in vivo*,* three xenograft models were utilized: Rat1-myr-p110α, Rat1-myr-p110δ, and HBRX2524. For Rat1-myr-p110α and Rat1-myr-p110δ, Rat1 fibroblasts were transfected with an N-terminal myristoylated form of PI3Kα or PI3Kδ isoform, which led to constitutive activation of the PI3K pathway. [13] Tumor xenografts were then grown subcutaneously in nude mice or nude rats by injection of 2 to 3 × 10^6^ cells into the right flank (for Rat1-myr-p110α and Rat1-myr-p110δ). For HBRX2524, a patient-derived breast cancer tumor bearing the H1047R *PIK3CA* activating mutation was established by subcutaneously implanting resected patient tumor samples in nude mice without any in vitro manipulations. CLR457 antitumor activity was then evaluated as described previously. [13] Systemic exposure and bioavailability of CLR457 and effects on glucose homeostasis were assessed after single and repeat dosing in mice and rats. Plasma insulin levels were assessed with a commercially available enzyme-linked immunosorbent assay (ELISA) kit (Mercodia). Blood glucose levels were determined using a Glucometer (One Touch Ultra®, LifeScan). (Supplemental Methods).

### Clinical study design

This was a first-in-human multicenter, open-label, phase I study investigating CLR457 in adult patients with PI3K-activated advanced solid tumors (NCT02189174). The primary objective was to define the maximum tolerated dose (MTD). Secondary objectives included assessments for safety, tolerability, pharmacokinetic (PK) profile, and preliminary antitumor activity. All procedures performed involving human participants were in accordance with the ethical standards of the institutional and/or national research committee and with the 1964 Helsinki declaration and its later amendments or comparable ethical standards. Informed consent was obtained from all individual participants included in the study.

### Patients

Patients had histologically documented locally advanced or metastatic solid tumors harboring an activating *PIK3CA* mutation or amplification, *PTEN* loss of function, c-MET activation, [16, 17] EGFR activation,^17^ and/or HER2 overexpression,^17^ or endometrial cancer [18] that had progressed or failed standard therapy. Other key inclusion criteria included age ≥ 18 years, Eastern Cooperative Oncology Group performance status (ECOG-PS) ≤2, adequate organ function, measurable or non-measurable disease as determined by Response Evaluation Criteria in Solid Tumors (RECIST) version 1.1. [19] Key exclusion criteria included type 1 or 2 diabetes mellitus requiring insulin treatment, fasting plasma glucose ≥140 mg/dL (7.8 mmol/L), prior pancreatitis, pneumonitis, active small or large intestinal inflammation, central nervous system (CNS) metastasis, prior treatment with AKT, mTOR, and PI3K inhibitors, or inadequate cardiac, renal, lung or liver organ function.

### Study drug and treatment

CLR457 was administered orally once daily (QD) on a continuous schedule until patients experienced unacceptable toxicity, had progressive disease, and/or treatment was discontinued at the discretion of the investigator or withdrawal of consent. The starting dose for CLR457 was 5 mg QD, based on preclinical toxicology studies in accordance with ICH guidelines, and was administered at the same time each day ±2 h in a fasted state. Various doses were planned up to 300 mg QD until MTD determination.

### Safety assessment

Safety was evaluated by incidence, nature, severity, and relatedness of adverse events (AEs), and graded according to National Cancer Institute - Common Terminology Criteria for Adverse Events (NCI CTCAE) v4.0.3. DLTs were defined as any hematologic or non-hematologic ≥ Grade 3 AE assessed as possibly related to CLR457 that occurred within the first cycle (28 days) of treatment. In addition, uncontrolled (> 14 days) Grade 2 rash or hyperglycemia were also considered DLTs. All AEs regardless of attribution were collected until 30 days following the last administration of treatment or study discontinuation/termination.

### Mutational and pharmacokinetic analysis

Pretreatment tumoral mutational analysis was performed at the treating institutions and reviewed by the Investigator. CLR457 PK was evaluated using plasma samples collected during Cycle 1 - Day 1 (C1D1) and Cycle 1 - Day 15 (C1D15) at pre-dose, and 0.5, 1, 2, 3, 4, 6, 8, 12, and 24 h post-dose by liquid chromatography–mass spectrometry (LC-MS). PK parameters including area under the curve (AUC), maximum serum concentration (C_max_), and time taken to reach C_max_ (T_max_) were calculated using non-compartmental methods in Phoenix WinNonlin. [20]

### Efficacy assessments

Efficacy assessments including overall response rate (ORR) and disease control rate (DCR) were analyzed as per RECIST v1.1 by local investigator interpretation based on interval imaging every 2 cycles. [19]

### Statistical methods

For the preclinical studies, absolute values for primary tumor growth and body weight were used to make the statistical comparisons between groups (one way analysis of variance [ANOVA] test followed by Dunnett’s test for normally distributed data; ANOVA on ranks for not normally distributed data followed by Dunnett’s test for equal group size or Dunn’s for unequal group size). The significant level was set at *p* < 0.05.

Descriptive methods were used to tabulate toxicity and response endpoints in the clinical trial. All patients treated with CLR457 were included for safety. The MTD was defined as the highest dose of CLR457 not causing a DLT in >33% of patients during the first treatment cycle. Dose escalation was guided by an adaptive Bayesian logistic regression model (BLRM) incorporating the escalation with overdose control (EWOC) principle. [21] Under EWOC, the dose selected as the MTD must have a < 25% chance that the true DLT rate exceeds 33%, given the available DLT information. The study data (including DLTs during cycle 1 and other safety and PK data) were reviewed by the sponsor and trial investigators at each dose level. Patients had to complete a minimum of one cycle of treatment with the minimum safety evaluation and drug exposure or to have had a DLT within the first cycle of treatment to be considered evaluable for dose escalation decisions. The Full Analysis Set (FAS) included all patients who received at least one dose of CLR457. Safety set included all patients who received at least one dose of CLR457 and had at least one valid post-baseline assessment. The Dose-determining set (DDS) included all patients from the safety set who either met the following minimum exposure criterion and had sufficient safety evaluations, or experienced a DLT during Cycle 1 (the first 28 days of dosing). The Pharmacokinetic analysis set included all patients who had at least one blood sample providing evaluable PK data.

The ORR was defined as the proportion of patients at each post-baseline scan who exhibited a complete response (CR) or partial response (PR) according to RECIST 1.1. The DCR was defined as the proportion of patients at each post-baseline scan who exhibited a CR, PR or stable disease (SD).

## Results

### Preclinical characterization of CLR457

The IC_50_ of CLR457 against the different PI3K isoforms was as follows: p110α: 89 ± 29 nM; p110β: 56 ± 35 nM; p110δ: 39 ± 10 nM; p110γ: 230 nM ± 31 nM. In vitro profiling studies demonstrated that CLR457 also inhibited the most common forms of *PIK3CA* mutant isoforms: E545K (helical domain mutation) and H1047R (kinase domain mutation) (data not shown).

**Fig. 1:**
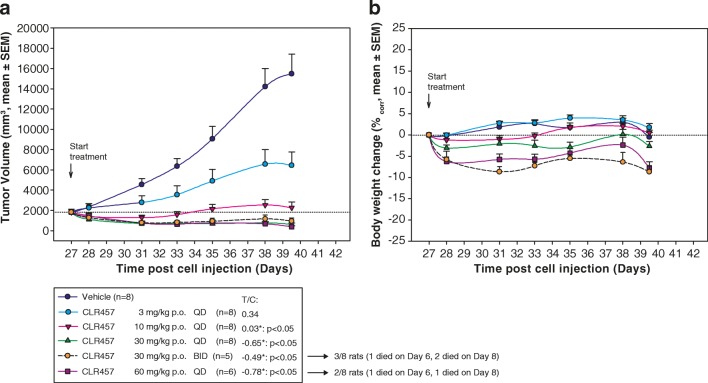
Antitumor activity (**a**) and effect on body weight (**b**) of CLR457 against Rat1-myr-p110α tumors grown in nude rats

To characterize the in vivo antitumor activity of CLR457, xenograft nude rats and mice were treated with CLR457. CLR457 administered orally at doses of 3, 10, 30, and 60 mg/kg QD and 30 mg/kg twice daily (BID) demonstrated dose proportional antitumor activity against Rat1-myr-p110α in nude rat xenografts (Fig. 1a, b). Tumor regression was observed at 30 mg/kg QD and higher. Body weight did not fluctuate more than ±5% for most tested doses. Similar antitumor efficacy and weight effects were observed in murine xenograft models with tumor regression occurring at 20 mg/kg BID (Rat1-myr-p110α, Rat1-myr-p110δ, and HBRX2524) (Supplemental Fig. 1A-C).

At doses where tumor regression was observed, the corresponding steady state AUC was 22,769 ng*h/mL. The mean tissue/plasma exposure ratio of CLR457 based on AUC measured in brain, skin, spleen, eyes, pancreas and heart was 0.07, 1, 1.22, 0.51, 1.74 and 0.83, respectively, and indicated minimal central nervous system (CNS) penetration.

**Fig. 2:**
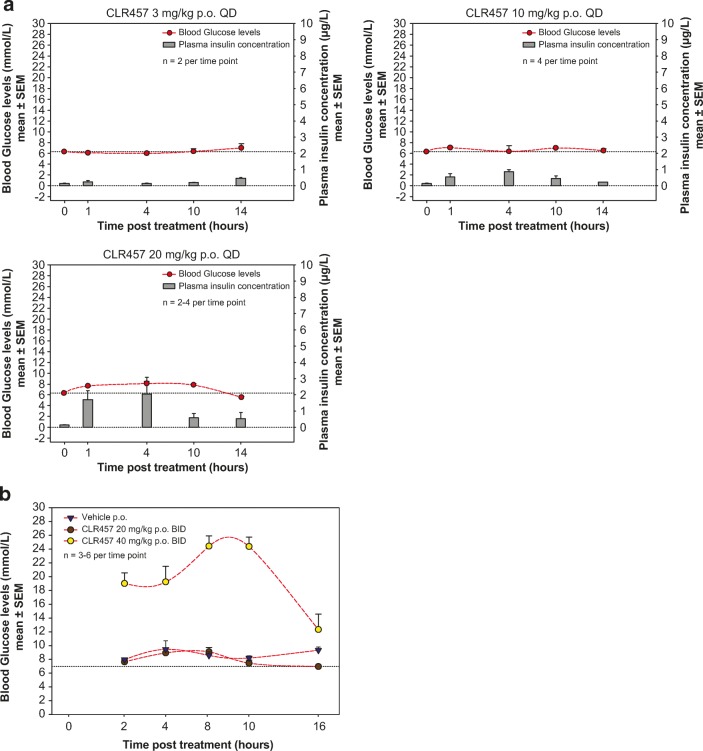
**a** Effect of increasing doses of CLR457 over time on blood glucose and insulin levels in Rat1-myr-p110α tumor bearing nude mice (QD dosing). **b** Effect of CLR457 over time on blood glucose and insulin levels in Rat1-myr-p110α tumor bearing nude mice (BID dosing)

Blood glucose and plasma insulin levels were assayed in mice (Fig. 2a, b) and rats (Supplemental Fig. 2) treated with CLR457. [22–24] Plasma insulin levels increased proportionally to CLR457 concentration, and glucose levels were moderately perturbed at the dose necessary for tumor regression.

### Patient characteristics

A total of 31 patients were enrolled in the dose escalation phase from August 2014 to November 2015 (Table 1). The median age of patients was 61 years (range, 42–80 years), and ECOG-PS status was 0 (38.7%) or 1 (61.3%). The most common cancers were endometrium (6 patients; 19.4%), breast (5 patients; 16.1%), and colon (4 patients, 12.9%). Activating *PIK3CA* mutations (19 patients, 61.3%) followed by loss of *PTEN* (6 patients, 19.4%) were the most common alterations. Patients received CLR457 orally once daily at doses ranging from 5 to 100 mg. The median duration of exposure was 7.1 weeks. The median actual dose intensity was 44.4 mg/day (Table 2).

**Table 1:** Patient demographics and baseline characteristics

	All Patients, *N* = 31
Median age (range)	61 (42–80)
Sex n (%)	
Female	23 (74.2)
Male	8 (25.8)
Race n (%)	
Asian	9 (29.0)
Caucasian	22 (71.0)
ECOG PS n (%)	
0	12 (38.7)
1	19 (61.3)
Primary site of cancer, n (%)	
Endometrium	6 (19.4)
Breast	5 (16.1)
Colon	4 (12.9)
Bladder	2 (6.5)
Cervix	2 (6.5)
Lung	2 (6.5)
Ovary	2 (6.5)
Thyroid	2 (6.5)
Esophago-gastric junction	1 (3.2)
Gall bladder	1 (3.2)
Left submandibular gland	1 (3.2)
Oral cavity	1 (3.2)
Stomach	1 (3.2)
Uterus	1 (3.2)
Mutational Status, n (%)*	
*PI3KCA* mutant	19 (61.3)
*PTEN* mutant	6 (19.4)
*ERBB2* amplified or over-expressed	3 (9.7)
*EGFR* mutant	1 (3.2)
*c-MET* amplified	1 (3.2)

*Due to co-occurring mutations, total may not summate to 100%

**Table 2:** Patient disposition, DLTs, duration of exposure and dose intensity

	CLR457 5 mg	CLR457 10 mg	CLR457 20 mg	CLR457 40 mg	CLR457 70 mg	CLR457 100 mg	All Patients
Patient disposition (FAS)	*N* = 2	*N* = 3	*N* = 4	*N* = 5	*N* = 6	N = 11	*N* = 31
Patients treated, n (%)	2 (100)	3 (100)	4 (100)	5 (100)	6 (100)	11 (100)	31 (100)
Treatment discontinued, n (%)	2 (100)	3 (100)	4 (100)	5 (100)	6 (100)	11 (100)	31 (100)
Primary reason for treatment discontinuation							
Adverse event, n (%)	0	0	0	1 (20.0)	0	4 (36.4)	5 (16.1)
Physician decision, n (%)	0	0	0	0	0	2 (18.2)	2 (6.5)
Progressive disease, n (%)	2 (100)	3 (100)	4 (100)	3 (60.0)	6 (100)	3 (27.3)	21 (67.7)
Withdrawal of consent, n (%)	0	0	0	1 (20.0)	0	2 (18.2)	3 (9.7)
Duration of exposure and dose intensity (Safety set)	N = 2	N = 3	N = 4	N = 5	N = 6	N = 11	N = 31
Duration of exposure, median (range), weeks	5.93 (4.0–7.9)	7.14 (3.7–8.0)	7.86 (6.0–8.0)	10.0 (3.9–15.6)	6.07 (4.1–16.0)	6.29 (1.4–13.9)	7.14 (1.4–16.0)
Actual dose intensity, median (range)	5.00 (5.0–5.0)	10.00 (10.0–10.0)	20.00 (19.5–20.0)	40.00 (29.7–40.0)	66.67 (44.4–70.0)	80.00 (41.4–100.0)	44.38 (5.0–100.0)
DLTs occurring in the first cycle (DDS)	N = 2	N = 3	N = 4	N = 5	N = 6	*N* = 9	*N* = 29
Total DLTs, n (%)	0	0	0	1 (20.0)	0	3 (33.3)	4 (13.8)
Hyperglycemia, n (%)	0	0	0	0	0	1 (11.1)	1 (3.4)
Maculo-papular rash, n (%)	0	0	0	1 (20.0)	0	2 (22.2)	3 (10.3)

*DDS*, dose determining set; *DLT*, dose limiting toxicity; *FAS*, full analysis set

### MTD determination and safety

Of the 31 patients treated with CLR457, DLTs were reported in 4 patients. Among these DLTs, Grade 3 hyperglycemia was reported in 1 patient dosed at 100 mg and maculo-papular rash was observed in 3 patients, one at 40 mg and two at 100 mg, respectively (Table 2). As 3 of 11 (27.2%) patients in the 100 mg cohort experienced a DLT, the BLRM model theoretically would allow further dose escalation. The overall toxicity profile described below; however, lead the study Investigators and Sponsor to terminate the study. The MTD was therefore not determined.

All patients were evaluable for safety (Table 3). The most frequent study drug-related AEs of any grade included stomatitis (45.2%), diarrhea (38.7%), maculo-papular rash (35.5%), fatigue and nausea (29% each), decreased appetite and hyperglycemia (22.6% each). Grade 3/4 drug-related AEs were observed in 51.6% of patients. Maculo-papular rash (25.8%) was the most common grade 3/4 AE in ≥10% of patients. Five patients (16.1%) experienced drug-related AEs leading to discontinuation of study treatment, rash being the most common. AEs requiring dose interruptions or dose reductions were reported in 18 (58.1%) patients, the most frequent being maculo-papular rash (8 patients, 44.44%) and diarrhea (4 patients, 22%). SAEs suspected to be related to the study treatment were reported in 7 patients (22.6%). Colitis, diarrhea, and pneumonitis (6.5% each) were the most frequently reported SAEs. At the 100 mg dose, 7 of 11 pts. (63.6%) reported at least one SAE irrespective of treatment during the study occurring outside the 28 day DLT window, notably 1 case of colitis and 2 cases of pneumonitis. Six on-study deaths occurred, 5 due to radiographic disease progression and 1 due to multi-organ failure, however none were attributed to the study drug.

**Table 3:** Suspected study drug-related adverse events (any grade in >5% of patients)

Preferred Term	CLR457 5 mg N = 2 n (%)		CLR457 10 mg N = 3 n (%)		CLR457 20 mg N = 4 n (%)		CLR457 40 mg N = 5 n (%)		CLR457 70 mg N = 6 n (%)		CLR457 100 mg N = 11 n (%)		All Patients *N* = 31 n (%)	
	All Grade	Grade ≥ 3	All Grade	Grade ≥ 3	All Grade	Grade ≥ 3	All Grade	Grade ≥ 3	All Grade	Grade ≥ 3	All Grade	Grade ≥ 3	All Grade	Grade ≥ 3
Stomatitis	0	0	0	0	0	0	3 (60.0)	0	2 (33.3)	0	9 (81.8)	0	14 (45.2)	0
Diarrhea	0	0	0	0	0	0	3 (60.0)	0	4 (66.7)	1 (16.7)	5 (45.5)	1 (9.1)	12 (38.7)	2 (6.5)
Maculo-papular rash	0	0	0	0	0	0	2 (40.0)	2 (40.0)	2 (33.3)	1 (16.7)	7 (63.6)	5 (45.5)	11 (35.5)	8 (25.8)
Fatigue	1 (50.0)	0	1 (33.3)	0	1 (25.0)	0	1 (20.0)	0	1 (16.7)	0	4 (36.4)	1 (9.1)	9 (29.0)	1 (3.2)
Nausea	0	0	1 (33.3)	0	0	0	2 (40.0)	0	2 (33.3)	0	4 (36.4)	0	9 (29.0)	0
Decreased appetite	0	0	0	0	0	0	1 (20.0)	0	2 (33.3)	0	4 (36.4)	0	7 (22.6)	0
Hyperglyc-emia	0	0	0	0	0	0	2 (40.0)	0	1 (16.7)	0	4 (36.4)	1 (9.1)	7 (22.6)	1 (3.2)
Rash	0	0	0	0	1 (25.0)	0	0	0	2 (33.3)	1 (16.7)	3 (27.3)	1 (9.1)	6 (19.4)	2 (6.5)
Pruritus	0	0	0	0	0	0	1 (20.0)	0	1 (16.7)	0	3 (27.3)	0	5 (16.1)	0
Vomiting	0	0	0	0	0	0	1 (20.0)	0	2 (33.3)	0	2 (18.2)	0	5 (16.1)	0
Dysgeusia	0	0	0	0	0	0	1 (20.0)	0	0	0	3 (27.3)	0	4 (12.9)	0
Pyrexia	0	0	0	0	0	0	0	0	1 (16.7)	0	3 (27.3)	0	4 (12.9)	0
Asthenia	1 (50.0)	0	0	0	0	0	1 (20.0)	0	0	0	1 (9.1)	0	3 (9.7)	0
Chills	0	0	0	0	0	0	0	0	0	0	2 (18.2)	0	2 (6.5)	0
Colitis	0	0	0	0	0	0	0	0	1 (16.7)	1 (16.7)	1 (9.1)	1 (9.1)	2 (6.5)	2 (6.5)
Cough	0	0	0	0	0	0	0	0	0	0	2 (18.2)	0	2 (6.5)	0
Dehydration	0	0	0	0	0	0	0	0	1 (16.7)	1 (16.7)	1 (9.1)	0	2 (6.5)	1 (3.2)
Dry mouth	0	0	0	0	0	0	0	0	1 (16.7)	0	1 (9.1)	0	2 (6.5)	0
Dry skin	0	0	0	0	0	0	0	0	1 (16.7)	0	1 (9.1)	0	2 (6.5)	0
Headache	0	0	0	0	0	0	0	0	1 (16.7)	0	1 (9.1)	0	2 (6.5)	0
Hypomagn-esaemia	0	0	0	0	0	0	0	0	1 (16.7)	0	1 (9.1)	0	2 (6.5)	0
Decreased neutrophil count	0	0	0	0	0	0	0	0	1 (16.7)	0	1 (9.1)	1 (9.1)	2 (6.5)	1 (3.2)
Pneumonitis	0	0	0	0	0	0	0	0	0	0	2 (18.2)	2 (18.2)	2 (6.5)	2 (6.5)
Weight decreased	0	0	0	0	0	0	1 (20.0)	0	1 (16.7)	0	0	0	2 (6.5)	0

### Pharmacokinetic analysis

**Fig. 3:**
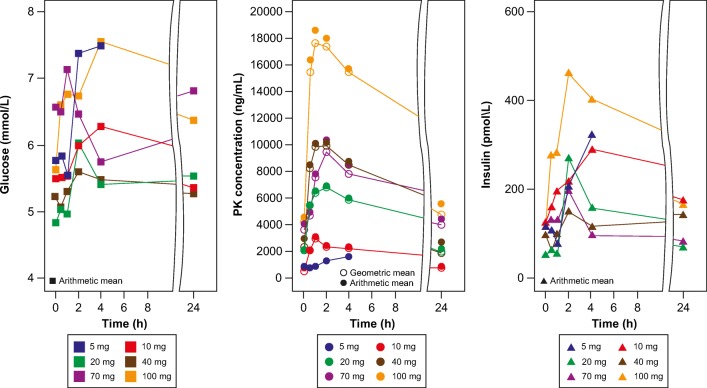
Time course of glucose, PK concentration and insulin in patients treated with CLR457

CLR457 was rapidly absorbed, with median T_max_ ranging between 1 to 3.5 h post dosing. CLR457 showed approximate dose-proportional pharmacokinetics on C1D1 as measured by AUC (0-24 h) and C_max_ across tested doses (Table 4). Following repeated daily dosing, overall exposure of CLR457 on C1D15 increased in a relatively proportional manner as measured by AUC_tau_ across tested doses, suggesting CLR457 exhibited linear PK even after multiple dosing. The geometric mean V/F and CL/F were similar across tested doses (V/F: range 43,139 to 115,503 mL; CL/F range 2610 to 4742 mL/h), and indicated low apparent clearance and low apparent volume of distribution. Steady state was reached by C1D15. Exposure at steady state (AUC_0-tau_ on Day 15) in humans following a daily dose of 100 mg was 23,754 ng*h/mL, which was similar to the exposure at which regression was observed in preclinical tumor xenograft models. The median elimination half-life (T_1/2_) of CLR457 was estimated from the observed accumulation ratio at steady state and ranged between 7.92 to 16.8 h (Table 5). At the dose of 100 mg QD, there was an increase in the pharmacodynamic markers glucose and insulin, consistent with that observed in the preclinical models, however this increase was not clinically significant (CTCAE grade ≤ 2) (Fig. 3) and was within the range of expected changes based on preclinical data (Fig. 2).

**Table 4:** Primary PK parameters for CLR457 at cycle 1 day 1 by treatment groups

Treatment	Statistics	AUC_(0-24h)_ (hr*ng/mL)	C_max_ (ng/mL)	T_max_ (hr)
CLR457 5 mg (N = 2)	n	2	2	2
	Geometric mean (CV%)	1182 (40.8)	100 (0.40)	
	Median (range)			2.50 (1.00–4.00)
CLR457 10 mg (N = 3)	n	3	3	3
	Geometric mean (CV%)	2241 (7.5)	230 (24.5)	
	Median (range)			1.00 (0.92–2.00)
CLR457 20 mg (N = 4)	n	4	4	4
	Geometric mean (CV%)	6718 (41.6)	475 (24.2)	
	Median (range)			2.51 (2.05–3.95)
CLR457 40 mg (N = 5)	n	5	5	5
	Geometric mean (CV%)	9567 (34.9)	687 (47.2)	
	Median (range)			2.07 (0.98–6.00)
CLR457 70 mg (N = 6)	n	6	6	6
	Geometric mean (CV%)	10,537 (47.6)	732 (44.8)	
	Median (range)			3.50 (0.72–24.1)
CLR457 100 mg (N = 11)	n	10	11	11
	Geometric mean (CV%)	18,390 (25.8)	1449 (28.7)	
	Median (range)			2.93 (0.50–7.53)

AUC_(0-24h)_, Area Under The Curve during 24 h; C_max_, maximum plasma concentration; T_max_, time to reach maximum (peak) plasma concentration following drug administration

**Table 5:** Primary PK parameters for CLR457 at cycle 1 day 15 by treatment groups

Treatment	Statistics	AUC_tau_ (hr*ng/mL)	CL/F (mL/h)	V/F (mL)	R_acc_	T_1/2_, acc (hr)
CLR457 5 mg (N = 2)	n	2	2	2	2	2
	Geometric mean (CV%)	1665 (81.7)	3003 (81.7)	43,139 (6.6)	1.4 (33.4)	
	Median (range)					13.9 (7.58–20.20)
CLR457 10 mg (N = 3)	n	3	3	3	3	3
	Geometric mean (CV%)	2846 (25.5)	3514 (25.5)	66,520 (11.5)	1.3 (24.3)	
	Median (range)					7.92 (6.53–18.40)
CLR457 20 mg (N = 4)	n	4	4	4	4	3
	Geometric mean (CV%)	7662 (45.3)	2610 (45.3)	55,193 (32.1)	1.1 (28.4)	
	Median (range)					11.8 (5.71–15.34)
CLR457 40 mg (N = 5)	n	5	5	5	5	4
	Geometric mean (CV%)	11,727 (39.0)	3411 (39.0)	115,503 (109.2)	1.3 (38.4)	
	Median (range)					11.0 (4.13–27.90)
CLR457 70 mg (N = 6)	n	4	4	4	5	4
	Geometric mean (CV%)	14,762 (37.1)	4742 (37.1)	72,822 (49.9)	1.4 (29.5)	
	Median (range)					16.8 (11.9–23.70)
CLR457 100 mg (N = 11)	n	4	4	4	4	3
	Geometric mean (CV%)	23,754 (40.4)	4210 (40.4)	67,311 (38.2)	1.3 (37.0)	
	Median (range)					12.1 (5.04–27.0)

AUC_tau_, Area Under the Plasma Concentration-time Curve for a dosing interval; CL/F, apparent total clearance of the drug from plasma after oral administration; R_acc_, accumulation ratio; T_1/2_, acc, half-life for accumulation; V/F, apparent volume of distribution during terminal phase after non-intravenous administration

### Efficacy

Of 31 patients treated, 27 were evaluable for tumor response (Supplemental Table 1). No patient achieved a confirmed partial or complete response. Interestingly, a patient with ovarian carcinoma harboring a *PI3KCA H1074R* alteration, dosed at 100 mg daily, had a partial response with greater than 30% shrinkage of target lesions at first interval scan, though rash and colitis complicated further dosing and a partial response was not confirmed. The best objective response in evaluable patients was 8 with SD (25.8%), 2 with non-CR/non-PD (6.5%), and 17 with progression of disease (54.8%). The overall DCR was 32.3% (10 patients).

## Discussion

In summary, CLR457 is a potent pan-class I PI3K inhibitor with significant in vivo antitumor activity without CNS penetration. In the first-in-human study, we investigated the MTD, safety, PK, and efficacy of CLR457 in advanced PI3K-dependent solid tumors. CLR457 was poorly tolerated due primarily to gastrointestinal and dermatological AEs and doses above 100 mg were not explored. The MTD was not determined. Modest insulin perturbations and hyperglycemia, on-target effects of p110α blockade, were also observed indicating target engagement. Pharmacokinetic modeling demonstrated a favorable PK profile and at 100 mg daily dosing, CLR457 exposure in human was similar to the exposure necessary to achieve tumor regression in the preclinical models. CLR457 exhibited no meaningful antitumor activity outside of transient disease stabilization at this dose in humans. Taken together, the narrow therapeutic index observed with continuous oral dosing of CLR457 lead to cessation of clinical development.

The preclinical experiments support that CLR457 is a potent pan-class I PI3 kinase inhibitor. In biochemical assays, CLR457 results in balanced isoform blockade that is similar to other pan-class I inhibitors, albeit with varying potency. [11, 25, 26] CLR457 antitumor activity was observed and confirmed in multiple tumor xenograft models with constitutively active PI3K pathway. [13, 27] Expected effects on glucose homeostasis were also observed in vivo and are the result of mobilization of glucose transporters through an Insulin Receptor-Insulin Receptor Substrate-PI3K pathway-dependent mechanism. [22–24, 27] Importantly, although insulin resistance developed rapidly at most tested doses, such effects were transient and not significantly perturbed at concentrations necessary for in vivo tumor regression. Finally, CLR457 showed limited CNS penetration. In sum, these data supported clinical evaluation of this compound in patients with advanced solid tumors with PI3K pathway addiction.

In the clinical study, the safety profile of CLR457 was consistent with that described previously for other pan-PI3K inhibitors. Common AEs included gastrointestinal toxicity, rash, fatigue and hyperglycemia. Colitis and pneumonitis occurred as delayed toxicity. These AEs, thought to be on-target effects of p110α and p110δ inhibition, provide clinical evidence of pan-PI3K inhibition by CLR457. [7] Importantly, mood alterations seen with pan-PI3K that cross the blood brain barrier were not observed, and likely reflect the favorable pharmacokinetic properties of the compound. Unfortunately, grade 3/4 treatment-related toxicities were frequent and required dose interruptions, dose reductions, and treatment discontinuation. Although intermediate dose levels between 70 mg and 100 mg could have been explored (Fig. 3), predicted exposures based on the preclinical models and low levels of efficacy led us to terminate the study. At exposures predicted to interfere with oncogenic PI3K pathways, on-target effects in normal tissue led to intolerable toxicity, preventing further dose escalation. One caveat to this conclusion is that unidentified off-target effects of CLR457 may have contributed to untoward toxicity. However, our data and emerging clinical data, suggest the therapeutic index of pan-PI3K inhibitors may curtail their ultimate clinical potential. [12, 14, 15, 28] Phase III trials of the combination of fulvestrant with or without buparlisib showed that the addition PI3K inhibitor improved outcomes in patients with hormone receptor positive breast cancer; however, toxicities in the buparlisib arm limited treatment duration and intensity. [14, 15] Pictilisib toxicity was similar, limiting efficacy observed in combination with an aromatase inhibitor in hormone receptor positive breast cancer. [28]

Although CLR457 produced intolerable toxicity, our data argue for the continued development of PI3K inhibitors through alternative strategies to improve efficacy and tolerability. Pharmacologic manipulation of pan-class I inhibitors, such as pulsatile dosing resulting in transient complete target inhibition or modification of the route of administration, may widen the therapeutic index of this compound class. [11] Selective targeting of specific PI3K isoforms is well-described and also appears to be a viable therapeutic approach. [7] Provocative preclinical data further indicate that novel schedules and dose titration of combination PI3K isoform blockade might reduce toxicity while improving efficacy. [29] Furthermore, selectively targeting AKT driven tumors appears to have promising anti-tumor activity. [30] Finally, integration of animal and human dose-exposure relationships using model-based approaches, as utilized effectively in this study, will be imperative to guide estimation of a bioactive yet safe dose of PI3K inhibitors.

## Conclusions

In preclinical studies, CLR457 demonstrated pan-PI3K inhibition and inhibited growth of tumor xenografts having a constitutively active PI3K pathway. However, in the first-in-human study, CLR457 was associated with poor tolerability and limited activity, thus preventing further development of this agent. Multiple strategies to improve the therapeutic index of agents blocking the PI3K pathway are under clinical evaluation.

## Electronic supplementary material

ESM 1 (DOCX 40 kb)

Supplementary Table 1 (DOCX 43 kb)

Supplementary Figure 1 (DOCX 499 kb)

Supplementary Figure 2 (DOCX 137 kb)
