# Supplementary material for: Characterization and phase I study of CLR457, an orally bioavailable pan-class I PI3-kinase inhibitor
Source: Invest New Drugs. 2018 Aug 3;37(2):271–81. doi: 10.1007/s10637-018-0627-4 (PMC6440935; doi:10.1007/s10637-018-0627-4)
Supplement: Supplementary file 1 — (DOCX 40 kb) [file 10637_2018_627_MOESM1_ESM.docx]

**SUPPLEMENTARY**

**Supplementary Methods: Animal Models**

All applicable international, national, and/or institutional guidelines for the care and use of animals were followed. Female nude Rowett rats Hsd: RH-Fox1rnu were obtained from Harlan (Netherlands) and female Hsd: Athymic Nude-nu CPB mice were obtained from Harlan Winkelmann, Germany.
