# Supplementary material for: Characterization and phase I study of CLR457, an orally bioavailable pan-class I PI3-kinase inhibitor
Source: Invest New Drugs. 2018 Aug 3;37(2):271–81. doi: 10.1007/s10637-018-0627-4 (PMC6440935; doi:10.1007/s10637-018-0627-4)
Supplement: Supplementary file 2 — (DOCX 43 kb) [file 10637_2018_627_MOESM2_ESM.docx]

**Supplementary Table 1. Response by treatment group**

|  | **CLR457**  **5 mg**  **N = 2**  **n (%)** | **CLR457**  **10 mg**  **N = 3**  **n (%)** | **CLR457**  **20 mg**  **N = 4**  **n (%)** | **CLR457**  **40 mg**  **N = 5**  **n (%)** | **CLR457**  **70 mg**  **N = 6**  **n (%)** | **CLR457**  **100mg**  **N = 11**  **n (%)** | **All Patients**  **N = 31**  **n (%)** |
| --- | --- | --- | --- | --- | --- | --- | --- |
| **Best overall response** | | | | | | | |
| **Complete response (CR)** | 0 | 0 | 0 | 0 | 0 | 0 | 0 |
| **Partial response (PR)** | 0 | 0 | 0 | 0 | 0 | 0 | 0 |
| **Non-CR/non-PD** | 0 | 0 | 0 | 1 (20.0) | 0 | 1 (9.1) | 2 (6.5) |
| **Stable disease (SD)** | 0 | 0 | 0 | 2 (40.0) | 2 (33.3) | 4 (36.4) | 8 (25.8) |
| **Progressive disease (PD)** | 2 (100) | 3 (100) | 4 (100) | 2 (40.0) | 3 (50.0) | 3 (27.3) | 17 (54.8) |
| **Not evaluable** | 0 | 0 | 0 | 0 | 1 (16.7) | 3 (27.3) | 4 (12.9) |
| **Overall response rate  (ORR: CR + PR)** | **0** | **0** | **0** | **0** | **0** | **0** | **0** |
| **Disease control rate  (DCR : CR + PR + SD + non-CR/non-PD)** | **0** | **0** | **0** | **3 (60.0)** | **2 (33.3)** | **5 (45.5)** | **10 (32.3)** |
