# Supplementary material for: Characterization and phase I study of CLR457, an orally bioavailable pan-class I PI3-kinase inhibitor
Source: Invest New Drugs. 2018 Aug 3;37(2):271–81. doi: 10.1007/s10637-018-0627-4 (PMC6440935; doi:10.1007/s10637-018-0627-4)
Supplement: Supplementary file 3 — (DOCX 499 kb) [file 10637_2018_627_MOESM3_ESM.docx]

**Supplementary Figure 1: Antitumor activity and effect on body weight in (A) Rat1-myr-p110α tumors, (B) Rat1-myr-p110δ tumors, (C) HBRX2524 human primary breast tumor grown in nude mice.**

**A**


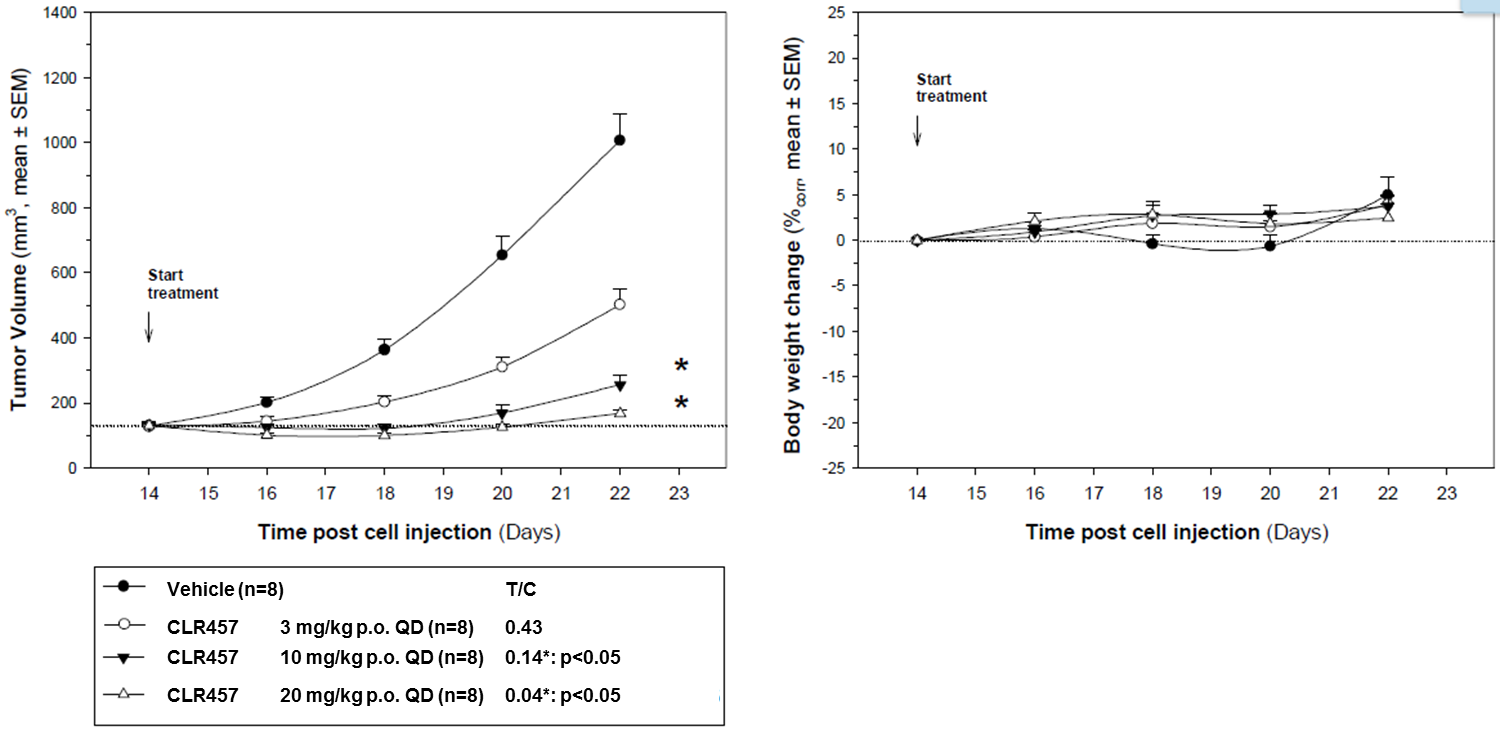


*p <0.05 vs. vehicle controls (ANOVA on ranks and post hoc Dunn’s test)

**B**

**
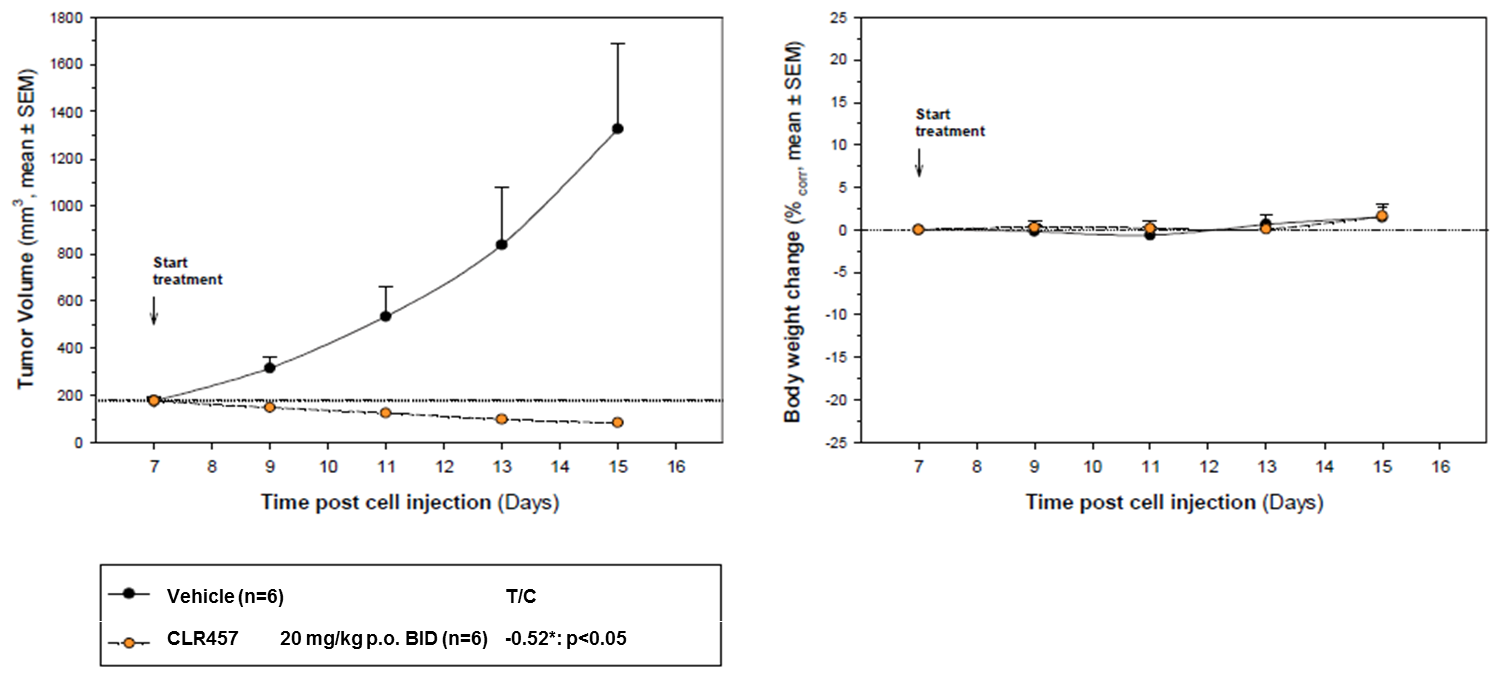
**

*p <0.05 vs. vehicle controls (ANOVA on ranks and post hoc Dunn’s test)

**C**


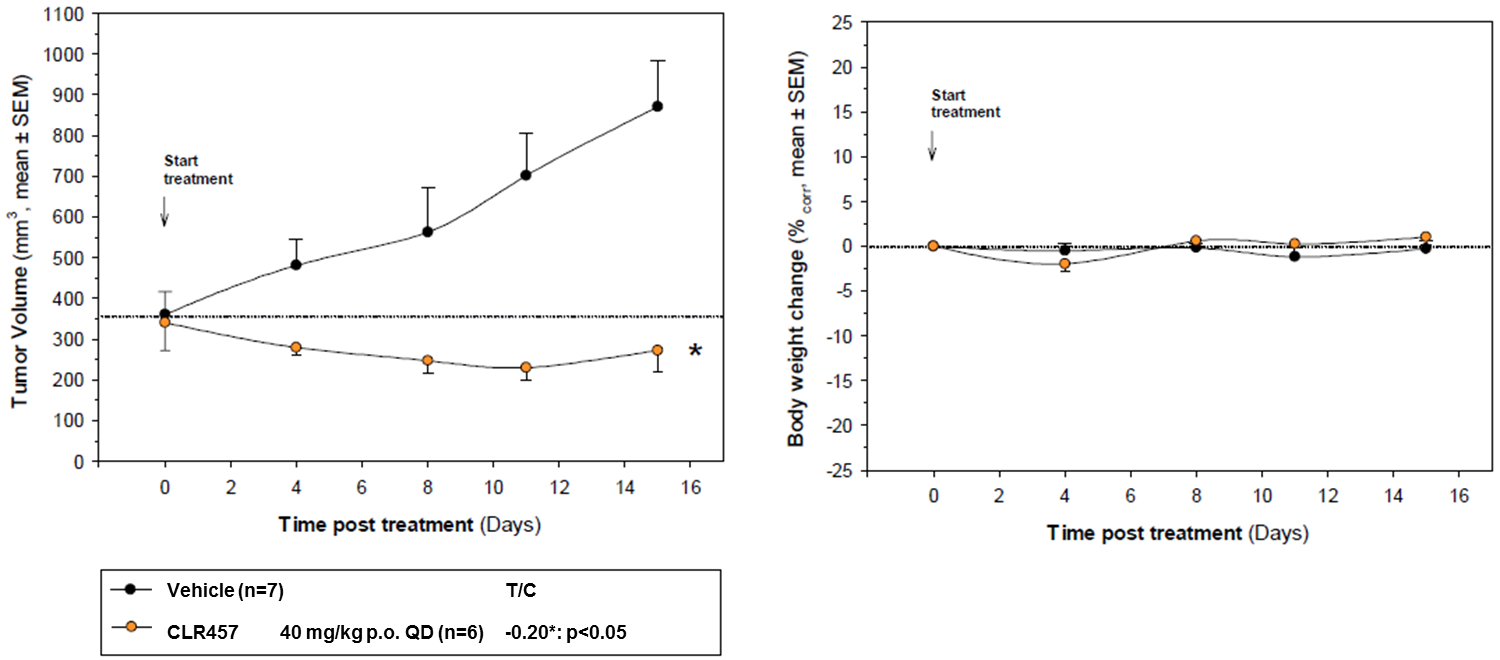


*p <0.05 vs. vehicle controls (ANOVA on ranks and post hoc Dunn’s test)
