# Supplementary material for: Characterization and phase I study of CLR457, an orally bioavailable pan-class I PI3-kinase inhibitor
Source: Invest New Drugs. 2018 Aug 3;37(2):271–81. doi: 10.1007/s10637-018-0627-4 (PMC6440935; doi:10.1007/s10637-018-0627-4)

**Supplementary Figure 2: Effect of CLR457 over time on blood glucose and insulin levels in Rat1-myr-p110α tumor bearing nude rats**


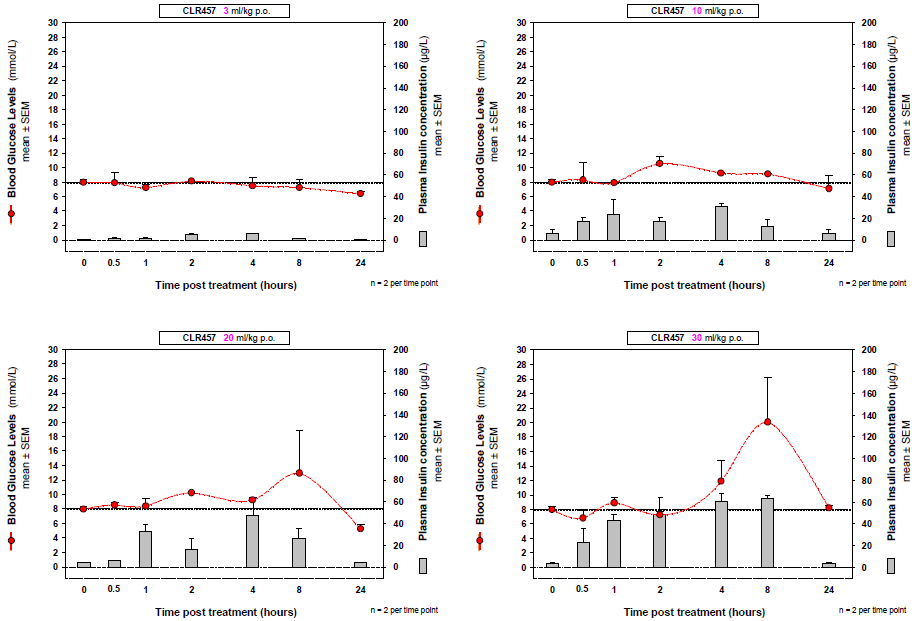

Supplement: Supplementary file 4 — (DOCX 137 kb) [file 10637_2018_627_MOESM4_ESM.docx]
